# The Brazilian Version of the Visual CARE 5Q: Instrument Translation and Cultural Adaptation

**DOI:** 10.1111/jep.70663

**Published:** 2026-10-08

**Authors:** Amanda Piovani Monteiro, Daniel Ferreira Dahdah, Regina Helena Vitale Torkomian Joaquim, Cristina Ortiz Sobrinho Valete

**Affiliations:** ^1^ Department of Psychology São Carlos Federal University São Carlos Brazil; ^2^ Pediatric Service, Hospital Universitário da UFSCar São Carlos Brazil; ^3^ Department of Occupational Therapy São Carlos Federal University São Carlos Brazil; ^4^ Department of Medicine São Carlos Federal University São Carlos Brazil

**Keywords:** adaptation, child, empathy, hospitalisation, translating

## Abstract

**Aim:**

To translate and cross‐culturally adapt the Visual CARE 5Q for use with Brazilian children.

**Design:**

A methodological cross‐sectional study.

**Methods:**

Portuguese translation and adaptation of the original Visual CARE 5Q. We followed five standardised steps: translation, synthesis, back‐translation, expert evaluation, and pilot testing. Ten expert judges and 25 hospitalised children aged 7−11 years participated in the study. Content validity was assessed using the Content Validity Index (CVI). Semantic, idiomatic, conceptual, and cultural equivalence were evaluated. Reliability was assessed using internal consistency, evaluated with Cronbach's alpha. The one‐factor structure was evaluated by exploratory factor analysis.

**Results:**

Adjustments for better comprehensiveness were necessary for items 1 and 5. All items reached full agreement after adjustments. Equivalence was adequate. Children evaluated the Visual CARE 5Q Brazilian version positively. The instrument demonstrated a unidimensional structure, consistent with the original version, and acceptable internal consistency (*α* = 0.73).

**Conclusion:**

After translation and cultural adaptation, the Visual CARE 5Q Brazilian version is applicable as a tool to assess hospitalised children's perception of clinical empathy in Brazil, contributing to the advancement of quality and child‐centered care.

**Impact:**

Evaluating children's perceived empathy during hospitalisation requires the use of instruments adapted to each country's reality and qualifies pediatric healthcare.

**Patient or Public Contribution:**

These results contribute to clinical practice by providing an instrument to evaluate perceived empathy among pediatric patients in Brazil.

## 1 Introduction

The doctor‐patient relationship is fundamental to the quality of healthcare, directly influencing patient satisfaction [1]. In this context, the Patient‐Centered Care (PCC) model proposes care that respects and responds to individual needs, values, and preferences, guiding clinical decision‐making [2]. Its implementation requires essential skills such as effective communication, active listening, empathy, and flexibility, which enable more individualised care [3]. Among these, empathy holds a prominent position in the medical sciences as a key resource for developing cognitive, affective, and social competencies essential to clinical practice [4]. Clinical empathy, also referred to as therapeutic empathy, can be understood as a process involving the following six interrelated core components: exploring, understanding, shared understanding, feeling, therapeutic action, and maintaining boundaries [5].

Given the impact of clinical empathy on treatment outcomes, instruments have been developed to assess it systematically, such as the Jefferson Scale of Empathy (JSE) and the CARE (Consultation and Relational Empathy) measure, both adapted and validated for the Brazilian context [6, 7]. However, these instruments were designed for healthcare professionals and adult patients, not pediatric patients. In this context, Visual CARE, a modified version of the original CARE scale, was designed for pediatric use. This scale includes three distinct questionnaires: (1) 5Q, for children aged 7 to 11 years; (2) 10Q, for adolescents aged 12 to 16 years; and (3) 10Q‐Parents, for parents of children aged 0 to 6 years or older children unable to respond independently [8]. This instrument has not yet been translated or culturally adapted for the Brazilian context.

This gap in the literature may be partially explained by the complexity involved in administering questionnaires to children, who often face difficulties in understanding questions, recalling relevant information, forming appropriate judgments, and communicating their responses, in addition to other context‐specific challenges [9]. Nevertheless, according to the United Nations, children, like all human beings, have the right to have their needs, values, and dignity respected [10]. To ensure this in hospital settings, they must be seen as patients, and their perspectives heard and valued [11].

Children can express their opinions on a range of issues, including their own healthcare, when supported and provided with appropriate tools [12, 13, 14]. The child's own perspective is particularly important, as it may differ significantly from that of third parties speaking on their behalf [15]. Considering this, it is essential to assess children's perspectives through questionnaires or evaluation scales, especially in the context of clinical empathy [16]. This approach enables the understanding of whether children feel that healthcare professionals recognise their feelings, perceptions, and needs, communicate this understanding clearly, and act upon it to provide therapeutic, supportive, and child‐centered care [17]. The lack of validated instruments to assess clinical empathy from children's perspectives in Brazil needs to be overcome. This study aims to translate and culturally adapt the Visual CARE 5Q for the Brazilian pediatric hospital context.

## 2 Method

### 2.1 Design

This is a methodological cross‐sectional study carried out between January 2026 and April 2026 in a pediatric ward of a public university hospital in Brazil. The Visual CARE 5Q was translated and adapted through five steps, after obtaining permission from the Talking Mats group. The steps carried out resulted in the CARE 5Q Brazilian version, and are presented in Figure 1.

**Figure 1:**
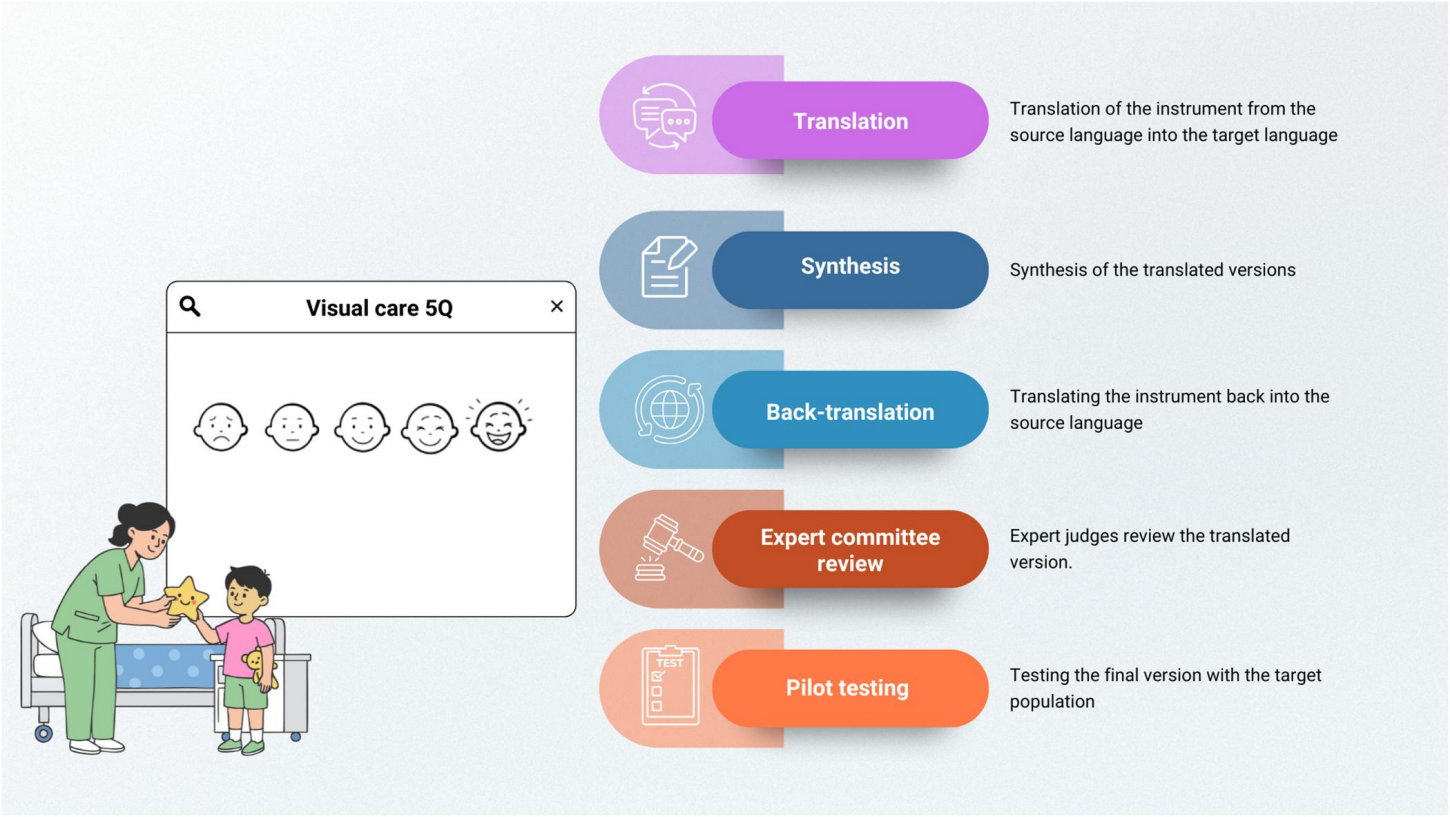
Steps of translation and cross‐cultural adaptation of the Visual CARE 5Q (Figure made with Canva).

### 2.2 The Original Visual CARE 5Q

The Visual CARE 5Q scale consists of five questions and uses simple, easy‐to‐understand language to measure relational empathy. Responses are recorded on a five‐point visual scale ranging from ‘poor’ (1 point) to ‘excellent’ (5 points). In the 5‐item version, only one missing or ‘not applicable’ item is allowed; in such cases, it should be replaced by the mean of the remaining items [18, 19]. The total score ranges from 5 to 25 for the 5‐item version, with higher scores indicating higher perceived clinical empathy [19].

In a previous study, the authors demonstrated that the items of both the 10Q/10Q‐Parent and the 5Q questionnaires were satisfactorily homogeneous, with an item‐total correlation higher than 0.75 for each item (range 0.76 to 0.89) [18].

### 2.3 Translation and Cultural Adaptation

We followed sequential steps: (1) translation of the instrument from the source language into the target language; (2) synthesis of the translated versions; (3) back‐translation; (4) expert committee review; and (5) pilot testing [20, 21, 22].

After identifying the most appropriate terms for the questionnaire, the versions were integrated into an initial version.

Ten judges were selected according to the following criteria: (a) knowledge in the area of empathy; (b) good command of the English language; and (c) prior experience in the process of cross‐cultural adaptation of instruments. They were selected by convenience, as members of the Center for Empathy in Healthcare in Brazil or specialists who work with the study coordinator, and were invited by email. After agreement to participate in the study they signed the informed consent and answered a REDCap questionnaire. The expert judges independently evaluated the comprehensiveness and relevance of the items (rated 1‐4) and the adequacy of the expressions to the Brazilian Portuguese context, as well as discussed any discrepancies, in order to obtain a preliminary version. The adjustments in the questionnaire were integrated into a final version. Two translators independently translated the instrument into Brazilian Portuguese. Thus, two independent Portuguese versions were obtained, and a synthesis of these two versions was developed. One of them had prior knowledge of the questionnaire's objectives due to being a member of the Center for Empathy in Healthcare in Brazil. The versions were reviewed, and no discrepancies were found between the original and the Brazilian version. This version was applied to a small group of hospitalised children aged between 7 and 11 years, applying the same inclusion and exclusion criteria of the next phase, to assess their views about clarity and comprehension of the items. As no difficulties in understanding were reported, the process advanced to the pilot study. At this stage, the instrument was administered to 25 hospitalised children between February 6, 2026, and March 17, 2026, through convenience sampling. The number of participants in the pilot phase was defined based on methodological recommendations suggesting samples of approximately 20‐30 individuals for this purpose [23]. The questionnaires were applied directly to children in the presence of a parent, during hospitalisation, in the morning or afternoon, through the electronic REDCap database. The researcher followed their responses and clarified any doubts when necessary.

Children and their parents were invited to participate voluntarily and consecutively as they were admitted to the pediatric ward. The selected participants met the following criteria: (1) aged 7‐11 years; (2) admitted to the pediatric ward and provided formal consent from their legal guardians, as well as their own assent. Exclusion criteria were as follows: (a) neurological or cognitive impairments; (b) any condition that could interfere with task comprehension; (c) those under sedation that could alter their level of consciousness.

Regarding the responses to the instrument, scores were assigned to each response category: 1 for ‘poor,’ 2 for ‘ok,’ 3 for ‘good,’ 4 for ‘very good,’ and 5 for ‘excellent.’ In cases where the option ‘does not apply’ was selected, the corresponding score was estimated as the mean of the participant's other responses and assigned to the respective item, in accordance with the guidelines of the Visual CARE [8]. Subsequently, the scores were summed for each participant, yielding a minimum possible score of 5 and a maximum of 25. Higher scores indicate a greater perception of care and empathy from healthcare professionals, whereas lower scores reflect a lower perception of clinical empathy.

The participants' characterisation questionnaires included age in months, reason for hospitalisation, length of hospital stay (days), presence of chronic illness (yes/no), admission to the intensive care unit during the current hospitalisation (yes/no), biological sex, the child's grade level (1st, 2nd, 3rd, 4th, 5th, 6th, or not currently studying) and whether the child lives with their parents (yes/no). The Visual CARE 5Q Brazilian version is in Figure 2 and in File S1.

**Figure 2:**
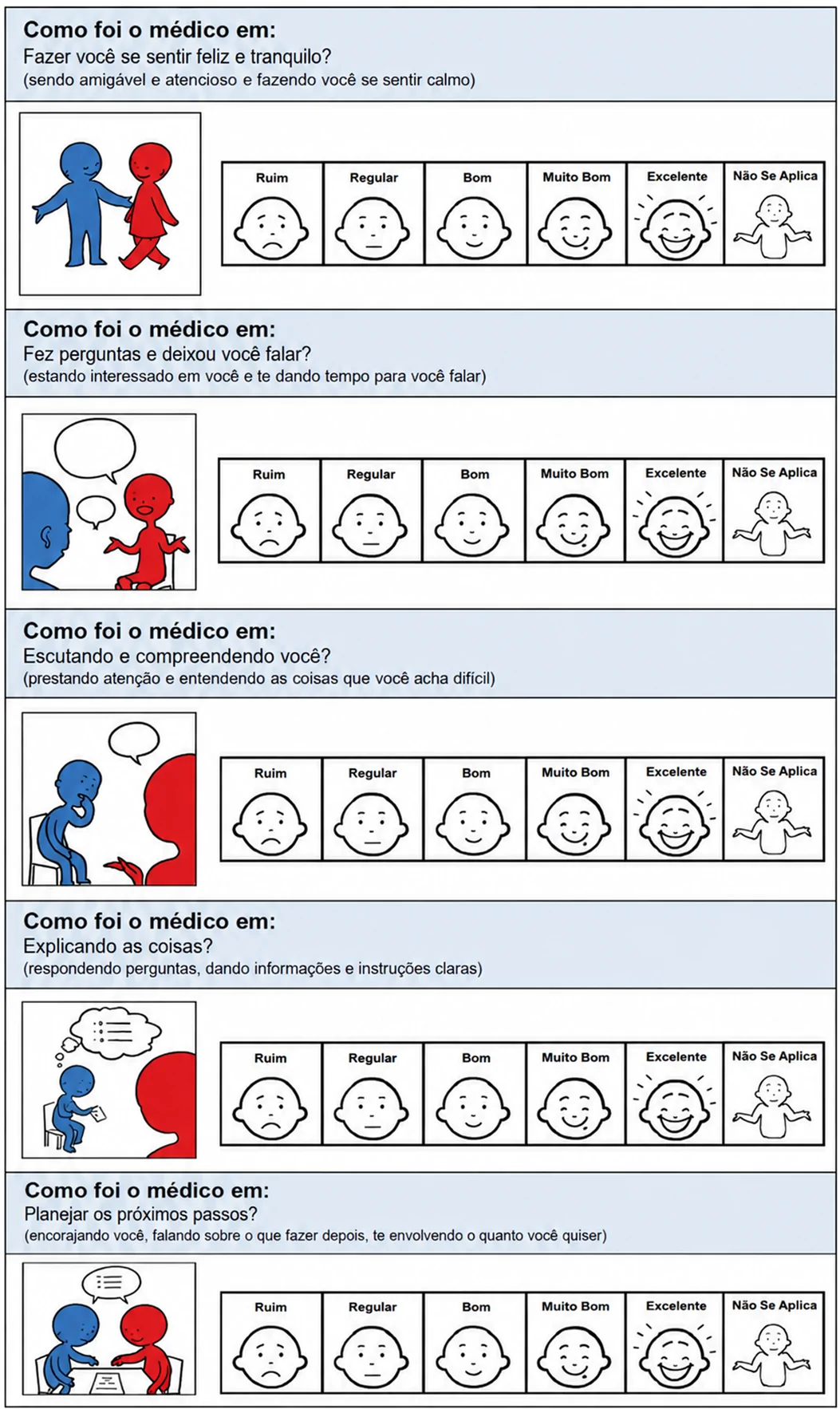
Final version of the Visual CARE 5Q Brazilian version.

### 2.4 Data Analysis

Children's and judges' characteristics were analysed by descriptive statistics, with medians, interquartile ranges, and proportions calculated. The following metric properties were analysed: item‐content validation index (I‐CVI) to measure the percentage of judges' agreement and internal consistency [24]. The I‐CVI allows the analysis of each item and the entire questionnaire. The calculation of this content validity was carried out by dividing the number of responses that were considered adequate (3 and 4) by the total number of responses for agreement: (1) comprehensiveness: an evaluation of clarity of the question; (2) relevance: how relevant is the question, and the following equivalences; (3) semantic: an evaluation based on grammar and vocabulary; (4) idiomatic: expressions that can lose their meaning in another language and need to be substituted; (5) conceptual: the evaluation of words with different conceptual meanings in different cultures; and (6) cultural: items that must correspond to those experienced in our cultural context.

Items with scores 1 or 2 were reviewed, adjusted, and submitted again to the judges until the I‐CVI was greater than 0.80 [25, 26, 27]. Two rounds were necessary. This step resulted in the final version used in step 5. Internal consistency of the CARE 5Q Brazilian version was evaluated. The responses of the 25 children included in this step were analysed, and Cronbach's alpha was calculated, considering the cutoff of 0.70 as good reliability [28].

This study did not aim to perform full psychometric validation of the questionnaire. We calculated correlations between the items by the Spearman test, and the construct validity was evaluated through exploratory analysis to confirm that only one factor was obtained. A principal component method was employed, with the Bartlett test, Cronbach alpha with 95% confidence interval (95% CI), and the Kaiser–Meyer–Olkin (KMO) test calculation. The Eigenvalues were calculated, and a Scree graph was generated. Data from the REDCap platform was exported to Stata version 19.0 for all the analyses.

## 3 Results

### 3.1 Judges' Characteristics

The panel of judges consisted of ten professionals, including 2 men and 8 women, aged between 28 and 60 years. The sample included physicians (*n* = 2; 20.0%), nurses (*n* = 1; 10.0%), psychologists (*n* = 2; 20.0%), occupational therapists (*n* = 3; 30.0%), and members of the Center for Empathy in Healthcare in Brazil (*n* = 2; 20.0%). Regarding academic background, 2 (20.0%) held an undergraduate degree, 2 (20.0%) had a postgraduate degree, and 6 (60.0%) had a master's or doctoral degree. Years since graduation ranged from 2 to 39 years. There was no missing data.

### 3.2 Content Validation

The item‐content validity index was calculated, adopting the 0.8 cutoff for agreement regarding relevance and comprehensiveness. Initially, agreement reached 1 for relevance for all items, and two items (1 and 5) showed less than 0.8 agreement for comprehensiveness (Table 1). For item 1, the suggestion was to change ‘relaxed’ to ‘calm.’ For item 5, the suggestion was to change ‘making a plan’ to ‘plan the next steps.’

**Table 1:** Initial agreement regarding relevance and comprehensiveness of the Visual CARE 5Q Brazilian version.

Agreement/Judges	Question 1		Question 2		Question 3		Question 4		Question 5	
	R	C	R	C	R	C	R	C	R	C
1	3	3	4	3	3	3	4	3	4	3
2	4	4	4	4	4	4	4	4	4	4
3	4	2	4	3	4	3	4	4	4	2
4	4	4	4	4	4	4	4	4	4	3
5	3	2	4	2	4	2	4	2	4	2
6	4	4	4	4	4	4	4	4	4	4
7	4	2	4	2	4	2	4	2	3	2
8	3	3	4	3	4	3	4	3	4	2
9	4	2	4	3	4	3	4	3	4	2
10	4	4	4	4	4	4	4	4	4	3
I‐CVI	1	0.6	1	0.8	1	0.8	1	0.8	1	0.5

Abbreviations: C, comprehensiveness; I‐CVI, item‐content validation index; R: relevance.

Subsequently, the judges' suggestions to improve comprehensiveness were incorporated, and the questionnaire was resubmitted for further evaluation. After these adjustments, agreement regarding comprehensiveness reached 1. The I‐CVI for the equivalence analysed are in Table 2.

**Table 2:** Semantic, idiomatic, conceptual, and cultural equivalence of the Visual CARE 5Q Brazilian version.

Equivalence/Judges	Semantic		Idiomatic		Conceptual		Cultural	
	1‐2	3‐4	1‐2	3‐4	1‐2	3‐4	1‐2	3‐4
1		x		x		x		x
2		x		x		x		x
3		x		x		x		x
4		x		x		x		x
5		x		x		x		x
6		x		x		x		x
7		x		x		x		x
8		x		x		x		x
9		x		x		x		x
10		x		x		x		x
I‐CVI		1		1		1		1

Abbreviation: I‐CVI, Item‐content validity index.

### 3.3 Children's Evaluation of the Questionnaire

The participants answered the five questions of the instrument and subsequently answered two additional questions concerning the comprehensiveness of the questionnaire and their perception of the study: (1) ‘Did you find the questions understandable?’; (2) ‘What did you think of the study?’

Children evaluated the Visual CARE 5Q Brazilian version positively. All respondents reported that the questions were understandable. Regarding their perception of the study, responses were mostly favourable, being described as ‘interesting,’ ‘very nice,’ ‘nice,’ and ‘good.’ Two participants found question 4 unusual and required additional explanation to answer it. Figure 3 presents these results.

**Figure 3:**
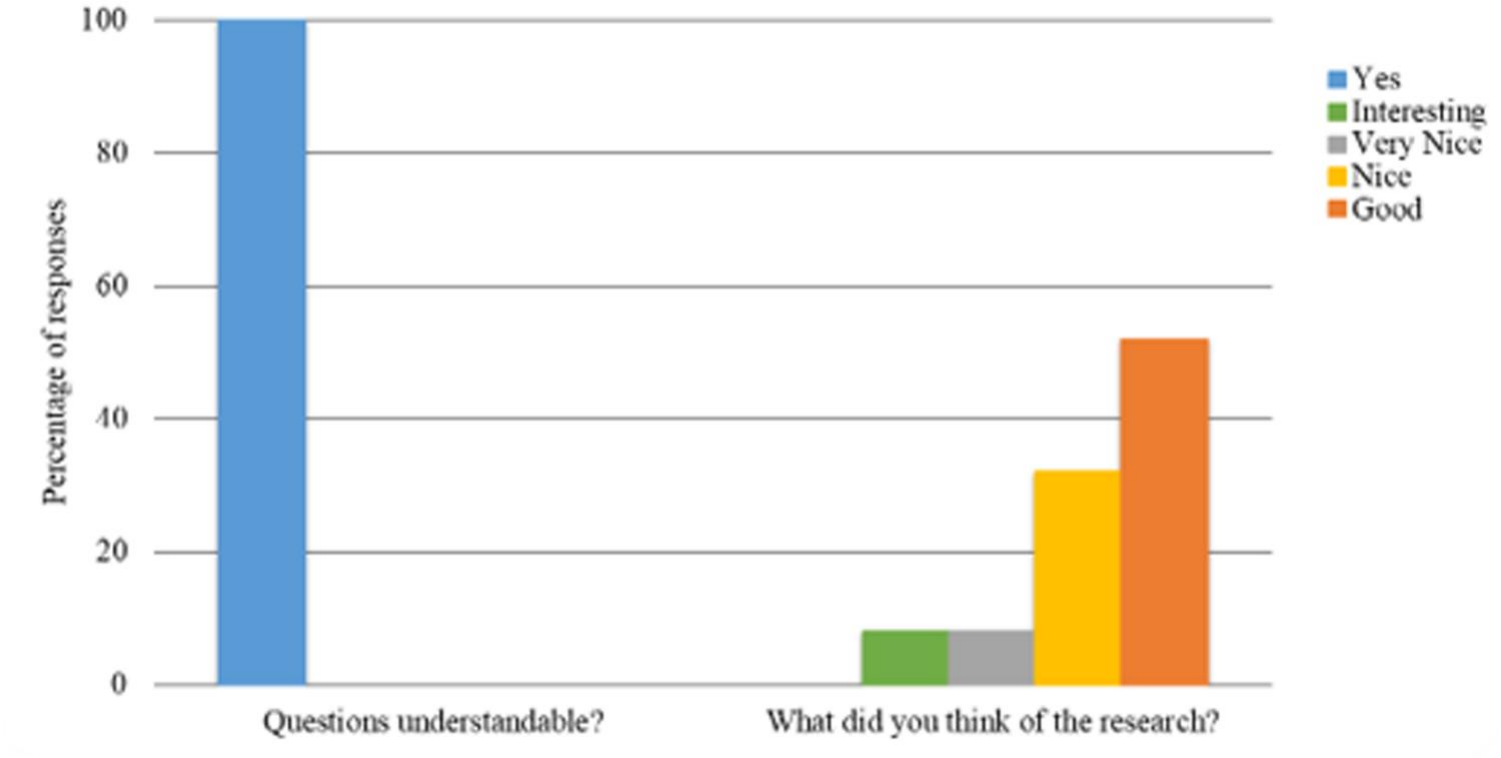
Children's evaluation of the Visual CARE 5Q Brazilian version.

The mean age was 8.6 years (SD = 1.59; range: 7–11 years), and the mean length of hospital stay was 3.1 days (SD = 4.63). Regarding sex, 16 (64%) were female, and 9 (36%) were male. The presence of chronic illness was reported in 3 (12%). As for the primary diagnoses, the most frequent were asthma in 12 (48%), appendicitis in 3 (12%), pneumonia in 2 (8%), renal problems in 2 (10%), mastoiditis in 1 (4%), epilepsy in 1 (4%), bronchitis in 1 (4%), diabetes in 1 (4%), meningitis in 1 (4%), and cardiac disease in 1 (4%). It was observed that 11 (44%) had a previous hospital admission. Regarding education, 6 (24%) were in the 2nd grade, 4 (16%) in the 3rd, 6 (24%) in the 4th, 1 (4%) in the 5th, and 8 (32%) in the 6th grade of elementary school. Concerning family context, 22 (88%) lived with their parents, and none were in institutional care. The average completion time of the scale was 2 min and 35 s. Considering the variability in age and reading skills, and in order to standardise the data collection procedure, the researcher read all items of the instrument aloud to each child, clarifying any questions during administration. The main questions were related to the response options of the scale (“ok,” “excellent,” and “does not not apply”). There was no missing data.

The results of the Visual CARE 5Q Brazilian version showed scores ranging from 13 to 25. The mean score was 19.2, suggesting an overall trend toward positive evaluations of perceived clinical empathy during care. The pilot results of the Visual CARE 5Q Brazilian version are presented in Table 3.

**Table 3:** Pilot results of the Visual CARE 5 Q Brazilian version.

Participant	Item 1	Item 2	Item 3	Item 4	Item 5	Total
**1**	3	5	3	3	3	17
**2**	5	4	4	5	4	22
**3**	5	4	3	4	5	21
**4**	3	4	5	3	5	20
**5**	5	5	4	5	5	24
**6**	3	2	3	1	4	13
**7**	3	3	3	4	3	16
**8**	3	5	5	3	4	20
**9**	3	2	3	3	4	15
**10**	4	3	5	4	5	21
**11**	5	5	4	1	4	19
**12**	5	5	4	5	5	24
**13**	5	5	5	5	5	25
**14**	3	3	3	2	4	15
**15**	4	5	5	5	5	24
**16**	3	3	3	3	3	15
**17**	4	1	3	3	4	15
**18**	3	4	3	2	4	16
**19**	3	3	3	3	3	15
**20**	3	3	5	3	5	19
**21**	1	5	1	4	5	16
**22**	4	5	4	5	5	23
**23**	3	4	4	5	5	21
**24**	5	5	4	5	4	23
**25**	5	4	5	4	5	23
**Mean (SD)**	3.7 (1.0)	3.8 (1.1)	3.7 (1.0)	3.6 (1.2)	4.3 (0.7)	19.2 (3.6)

Abbreviation: SD, Standard deviation.

### 3.4 Construct and Internal Consistency

The five items were correlated (Table 4). The principal components method revealed one factor (Eigenvalue: 2.46), and the Bartlett test was *X* ^2^ = 23.982; *p* = 0.008.

**Table 4:** Correlations between the Visual CARE 5Q Brazilian version items (*n* = 25).

	Item 1	Item 2	Item 3	Item 4	Item 5
**Item 1**	—				
**Item 2**	0.3730	—			
**Item 3**	0.4289	0.3420	—		
**Item 4**	0.5127	0.4908	0.3728	—	
**Item 5**	0.3349	0.3459	0.5556	0.5228	—

The Scree graph confirmed the one‐factor structure, considering an Eigenvalue > 1.0 (Figure 4), and the KMO test was 0.702. Cronbach's alpha was 0.7314 (95% CI: 0.5535–0.9092), and the model explained 49.3% of the total variance. The factorial loads were: item 1, 0.6979; item 2, 0.6438; item 3, 0.7136; item 4, 0.7361; item 5, 0.7160.

**Figure 4:**
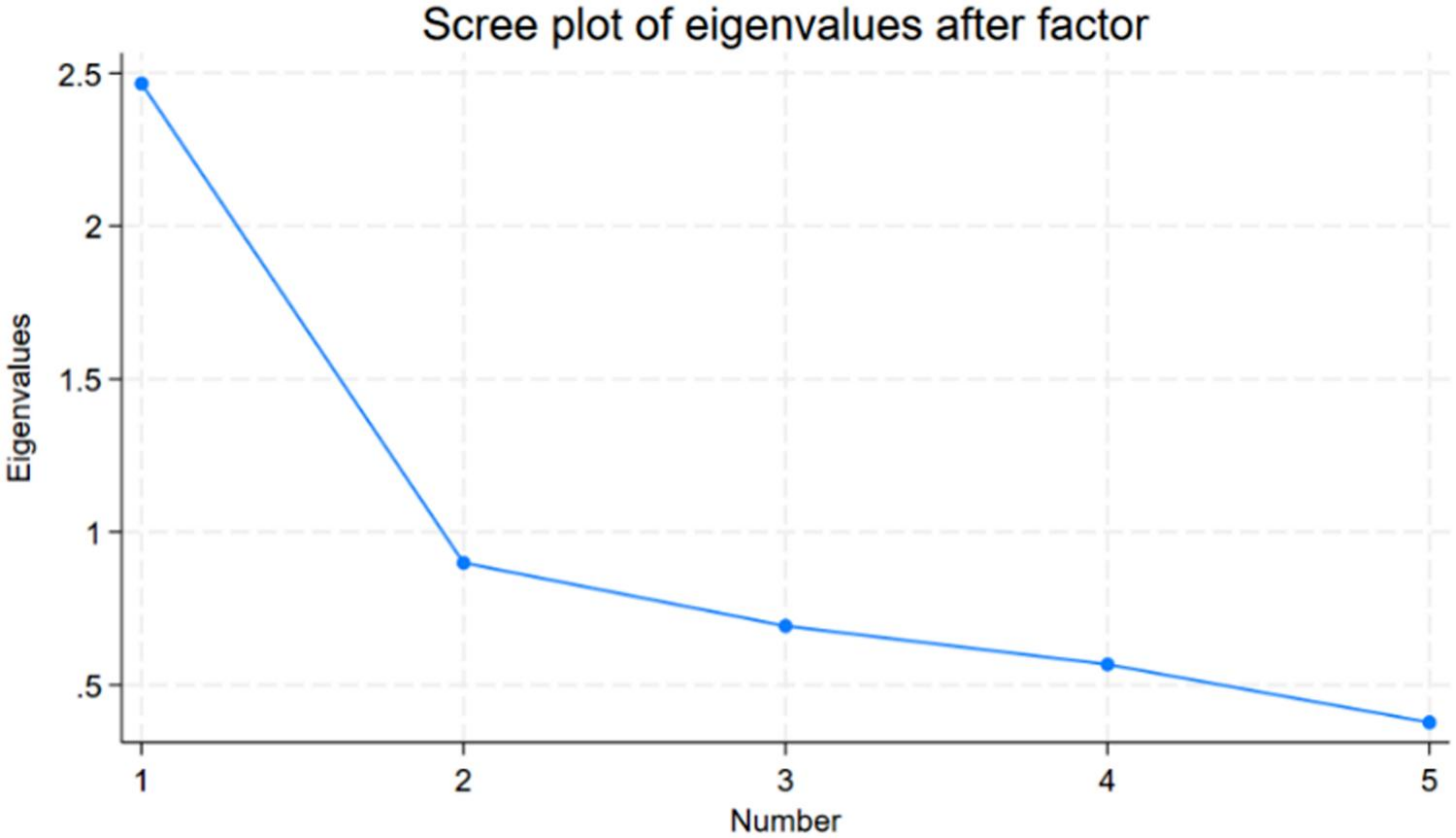
Scree plot of the explanatory factor analysis.

## 4 Discussion

This study was conducted to translate and culturally adapt the Visual CARE 5Q, aiming to make it available for use with hospitalised children in Brazil. The Visual CARE 5Q Brazilian version demonstrated good acceptance for children and Cronbach's alpha was 0.7314. One factor was identified in the factor analysis, and explained 49.3% of the total variance.

The adaptation process resulted in specific adjustments. Question 1 (*How was the doctor at: Making you feel happy and relaxed? being friendly and caring and making you feel calm*) and 5 (*How was the doctor at: Making a plan? encouraging you, talking about what to do next, involving you as much as you want*) needed to be adjusted to improve comprehensiveness. The terms ‘relaxed’ and ‘making a plan’ were adjusted and agreement regarding relevance and comprehensiveness of the items reached 1, and semantic, idiomatic, conceptual, and cultural equivalence was also 1. These values are considered adequate as suggested by literature [26].

In the Visual CARE 5Q Brazilian version, the observed I‐CVI for relevance and comprehensiveness was adequate, in accordance with the methodological guidelines recommended by another study [29]. The content validity of the original instrument and the Brazilian version was examined through distinct methodological approaches, reflecting the different epistemological perspectives adopted in each validation context. In the study of Place et al., content validity was inferred from the analysis of responses classified as ‘not applicable’; only 4.6% of the 87 participants who completed the Visual CARE 5Q version selected this option, indicating a high perceived relevance of the items among respondents [8]. Complementarily, face validity was investigated among healthcare professionals, of whom 95% considered the instrument relevant to their clinical practice and 90% deemed it useful evidence that reinforces its pertinence and applicability in the care setting. Although the evaluation strategies employed between studies are not directly comparable, given the distinct nature of the procedures involved, both studies converge in demonstrating high content validity of the instrument in their respective cultural and linguistic contexts. These results indicate that the Visual CARE 5Q Brazilian version is consistently perceived as relevant, comprehensible, and clinically pertinent in this cross‐cultural adaptation to Brazilian Portuguese.

Despite these findings, the data should be interpreted with caution, as children's responses in hospital settings may be influenced by social desirability effects. This occurs because they may tend to provide more positive evaluations of healthcare professionals due to the institutional context of care and the desire to meet perceived expectations [30, 31]. As a strategy to minimise this bias, data collection was conducted in the absence of healthcare professionals during instrument administration, and the researcher who collected the data was not part, reducing potential contextual pressures on the children's responses. However, it should be noted that no specific instrument was used to measure social desirability, which limits the ability to quantify the impact of this bias on the responses obtained.

Considering the participants' characteristics, some differences among other studies were observed. This study included children with a mean age of 8.6 years, a mean length of stay of 3.1 days, and 64% were female. Regarding the sample characteristics of other studies, Place et al. included a total of 109 respondents; among the children and adolescents who directly answered the instrument, most of them were male [8]. The study of Arigliani et al., in turn, included 416 participants, 221 males and 195 females, of whom 85 children aged 7 to 11 years completed the Visual CARE 5Q version [18]. In line with these findings, Wiljén et al. recruited 310 participants (146 males and 164 females), with 100 of them belonging to the 7–11 age group [19]. Regarding diagnostic profile, the most frequently reported conditions included in that study were oncological and haematological diseases, metabolic, renal, digestive, endocrine, and genitourinary conditions, as well as musculoskeletal, dermatological, neurological, and sensory conditions, different from the present study. Across these studies, different versions of the CARE Measure were employed according to the respondents' age group: the 10Q version, completed by parents or caregivers of children aged 0 to 6 years; the Visual CARE 5Q version, intended for children aged 7 to 11 years; and the Visual CARE 10Q version adapted for adolescents aged 12 to 18 years. In contrast, the present study was characterised by a smaller sample size, comprising 25 participants, nine males and 16 females, aged between 7 and 11 years. Therefore, direct comparability between the studies is considerably limited, as differences in the versions of the instrument used, the age ranges included, and the sample size impose constraints on generalisation and on the immediate comparison of the findings.

The construct of the Visual CARE 5Q Brazilian version revealed one factor, and the observed Cronbach alpha was 0.7314 with 49.3% of the total variance explained by the model, but we reinforce that the results of the factor analysis must be interpreted cautiously, specially due to the sample size. Arigliani et al. also observed in the exploratory factor analysis that all the items loaded into the first factor [18]. We consider the reliability observed in the present study acceptable. Place et al. observed a 0.74 Cronbach alpha in a study that investigated children's and supporters' ratings, close to the results observed in the present study [8]. Arigliani et al. observed a 0.88 Cronbach alpha for the Visual CARE 5Q in a study that included 85 children between 7 and 11 years of age [18]. Although the variation explained by the model was below the ideal threshold of 50%, this variance is marginal, accepted for brief assessment tools, and does not seem to compromise the use of the questionnaire. As suggested by hair et al., this empirical criteria must be balanced with the context being studied, especially in social and behavioural sciences [32]. Howard reviewed the reporting of factor analysis and suggested that cutoffs for one‐factor tests are too much high to discover problematic common method variance [33]. We must reinforce that we did not create a new questionnaire; we adapted an already validated one. We consider that these results reveal an acceptable consistency in the questionnaire, but should be interpreted cautiously.

## 5 Limitations of the Study

The sample consisted of 25 children. Although there is no defined consensus regarding sample size for factor analysis, we acknowledge this as a limitation given the variability of participants' characteristics. While the questionnaire presented acceptable consistency, future studies with larger, more diverse samples could further perform its full psychometric validation. Also, we did not measure, which is complex considering the construct of empathy test‐retest reliability. In addition, the profile of children included is acute disease, and thus, patients with chronic conditions were underrepresented. Finally, we did not assess possible social desirability effects; future studies could better investigate and measure this bias.

## 6 Conclusions

The present study described the translation and cultural adaptation process of the Visual CARE 5Q for Brazilian Portuguese for hospitalised children aged 7 to 11 years, demonstrating satisfactory results across the methodological steps adopted. Preliminary structural validity was observed, with full agreement among expert judges following adjustments. Exploratory factor analysis identified a single‐factor structure, consistent with the original construct of the scale. Internal consistency was considered acceptable, with a Cronbach's alpha of 0.73. Children evaluated the instrument positively, reporting adequate comprehension of the items and receptiveness to participation in the study. It is concluded that after translation and adaptation, the Visual CARE 5Q Brazilian version is applicable as a tool to assess hospitalised children's perception of clinical empathy in Brazil.

## Ethics Statement

This research was approved by the Institutional Research Ethics Committee (CAAE: 92257825.0.0000.5505). All the judges signed the informed consent form. Children and their legal guardians provided written informed consent before starting data collection. All of them were fully informed about the confidentiality and privacy of their information. Participation was strictly voluntary, and participants were assured of their right to withdraw at any time without providing a reason and without any penalty.

## Conflicts of Interest

The authors declare no conflicts of interest.

## Supporting information


Supporting File

## Data Availability

The data that support the findings of this study are available on request from the corresponding author. The data are not publicly available due to privacy or ethical restrictions.
