# Supplementary material for: The Brazilian Version of the Visual CARE 5Q: Instrument Translation and Cultural Adaptation
Source: J Eval Clin Pract. 2026 Oct 8;32(7):e70663. doi: 10.1111/jep.70663 (PMC13649088; doi:10.1111/jep.70663)
Supplement: Supplementary file 1 [file JEP-32-0-s001.docx]

**Supplementary material**

In this section, the final Portuguese version of the Visual CARE 5Q instrument is presented.

| Como foi o médico em… | Ruim   | Regular   | Bom   | Muito bom   | Excelente   | Não se aplica   |
| --- | --- | --- | --- | --- | --- | --- |
| Fazer você se sentir feliz e relaxado?  (ser amigável, atencioso e fazer você se sentir calmo)   |  |  |  |  |  |  |
| Fazer perguntas e deixar você falar?  (demonstrar interesse em você e dar tempo para se expressar)   |  |  |  |  |  |  |
| Ouvir e compreender?  (prestar atenção e conhecer as coisas que você considera difíceis)   |  |  |  |  |  |  |
| Explicar as coisas?  (responder perguntas, dar informações e instruções claras)   |  |  |  |  |  |  |
| Fazer um plano?  (encorajar você, conversar sobre os próximos passos e envolvê-lo(a) tanto quanto desejar)   |  |  |  |  |  |  |
